# Enhancing carrier transport and carrier capture with a good current spreading characteristic via graphene transparent conductive electrodes in InGaN/GaN multiple-quantum-well light emitting diodes

**DOI:** 10.1038/s41598-020-67274-1

**Published:** 2020-06-29

**Authors:** Shih-Wei Feng, Ying-Hsiang Wang, Chin-Yi Tsai, Tzu-Huan Cheng, Hsiang-Chen Wang

**Affiliations:** 1 Department of Applied Physics, National University of Kaohsiung No. 700, Kaohsiung University Road, Nan-Tzu Dist., 811, Kaohsiung City, Taiwan (R.O.C.); 2 LiveStrong Optoelectronics Cooperation, No. 82, Luke 5th Rd., Kaohsiung City, 821 Taiwan (R.O.C.); 3 Department of Mechanical Engineering and Advanced Institute of Manufacturing with High-tech Innovations, National Chung Cheng University, No. 168, Sec. 1, University Rd., Chia-yi, 621301 Taiwan (R.O.C.)

**Keywords:** Electrical and electronic engineering, Engineering, Physics, Applied physics, Optical physics

## Abstract

In this work, InGaN/GaN multiple-quantum-wells light-emitting diodes with and without graphene transparent conductive electrodes are studied with current-voltage, electroluminescence, and time-resolved electroluminescence (TREL) measurements. The results demonstrate that the applications of graphene electrodes on LED devices will spread injection carriers more uniformly into the active region and therefore result in a larger current density, broader luminescence area, and stronger EL intensity. In addition, the TREL data will be further analyzed by employing a 2-*N* theoretical model of carrier transport, capture, and escape processes. The combined experimental and theoretical results clearly indicate that those LEDs with graphene transparent conductive electrodes at *p*-junctions will have a shorter hole transport time along the lateral direction and thus a more efficient current spreading and a larger luminescence area. In addition, a shorter hole transport time will also expedite hole capture processes and result in a shorter capture time and better light emitting efficiency. Furthermore, as more carrier injected into the active regions of LEDs, thanks to graphene transparent conductive electrodes, excessive carriers need more time to proceed carrier recombination processes in QWs and result in a longer carrier recombination time. In short, the LED samples, with the help of graphene electrodes, are shown to have a better carrier transport efficiency, better carrier capture efficiency, and more electron-hole recombination. These research results provide important information for the carrier transport, carrier capture, and recombination processes in InGaN/GaN MQW LEDs with graphene transparent conductive electrodes.

## Introduction

III-nitrides have become the key material for light emitting diodes (LEDs), laser diodes, and solar cells^1–6^. Low resistance and transparent Ohmic contact to *p*-type GaN are crucial to improve current injection and light extraction efficiency of GaN-based LEDs^7–10^. Although device performance can be improved by using Indium Tin Oxide (ITO) used as a transparent conducting layer (TCL), the exclusive use of these oxides becomes problems such as cost, rarity supply of indium, and low optical transmittance in the ultraviolet wavelength regions^7–10^. One the other hand, graphene has been considered a promising material to replace ITO in GaN-based LEDs as a transparent conductive electrode^11–17^, because of its high transmittance in the UV region, carrier mobility, and conductivity. It has been demonstrated that the transparent 3D graphene foam was very effective in current spreading to enhance the performance of blue LEDs^11^. However, due to high sheet resistance and the large work function difference between graphene and *p*-GaN, the use of graphene as a TCL adversely increases the operating voltage of LEDs^7,11–14^. In addition, the poor adhesion of graphene on the devices also problematically results in a low current injection into LEDs^7^.

Electroluminescence (EL), output power, current-voltage (*I-V*) relationship, and quantum efficiency are the common measurements to characterize the device performance of GaN-based LEDs. Although device performance of GaN-based LEDs with graphene transparent conductive electrodes has been shown to be improved, these measurements cannot quantitatively describe carrier dynamic behaviors. Time-resolved electroluminescence (TREL) measurement under electrical fast pulse excitation is a power tool to explore the dynamic EL behaviors of carrier injection, carrier transport, carrier relaxation into active region, and carrier recombination at the excited states in active region of LEDs^18,19^. From the results of TREL measurements, *nitrogen*-polar InGaN/GaN LEDs with the opposite polarity and nonpolar *m*-plane InGaN/GaN LEDs with a low polarization effect were shown to provide the advantages of more carrier injection, transport, relaxation, and recombination^18,19^. However, the effects of graphene transparent conductive electrodes on the carrier dynamic behaviors of carrier injection, carrier transport, carrier capture into active region, and carrier recombination of InGaN/GaN LEDs with graphene transparent conductive electrodes are not well understood, and many important issues are yet to be studied.

This study will report the effects of graphene transparent conductive electrodes on the carrier transport, carrier capture, and recombination dynamics of InGaN/GaN MQW LEDs in comparison with those without graphene transparent conductive electrodes by using EL spectra, *I-V*, output power, and TREL measurements. The TREL data will be further analyzed by employing a 2-*N* theoretical model of carrier transport, capture, and recombination processes to investigate the effects of graphene transparent conductive electrodes on InGaN/GaN MQWs.

This paper is organized as follows: In Section 2, sample structures and experimental procedures are described. In Section 3, experimental results and discussions are reported. Finally, conclusions will be drawn in Section 4.

## Experiments

*I-V*, EL spectrum, and output power were measured with a source meter (Keithley 2614B), spectrometer (Ocean Optics, resolution 0.3 nm), calibrated integrating sphere, and power meter. Without an ohmic Ni/Au contact, an indium dot melting on the sample surface was used as a *n*-type contact for the probe station. For TREL measurement, a pulse generator (Tektronix AFG3152C) was used to generate 2.0–5.0 V, 0.5 μs pulse width, and 1 kHz repetition rate voltage pulses to the LEDs. The light output was focused and detected by a photosensor module containing a metal package PMT and a high-voltage power supply circuit (Hamamatsu H10721-210) operating directly on the surface of each LED. The same PMT voltages of the TREL measurement were applied for the LEDs. The parasitic capacitance in the test circuit would introduce the same RC delay for the two LEDs. The transit EL signals were recorded by a digital oscilloscope (Agilent DSO 6052 A) with a 500 MHz bandwidth. The overall resolution of TREL system is less than 2 ns. The detailed measurement was described in our previous study^18,19^.

Three InGaN/GaN MQW LEDs without graphene electrodes (denoted as LED_1_, LED_2_, and LED_3_ in this work) were prepared for the studies of device characteristics and carrier transport properties by using EL images, EL spectra, *I-V*, output power, and TREL measurements. Subsequently, graphene was transferred onto the *p*-GaN layer of these three InGaN/GaN MQW LEDs (denoted as LED_1G_, LED_2G_, and LED_3G_ correspondently). The same measurements were conducted on the LED_1G_, LED_2G_, and LED_3G_ samples. Device characteristics and carrier transport properties of InGaN/GaN MQW LEDs without and with multilayer graphene were compared and analyzed.

**Figure 1:**
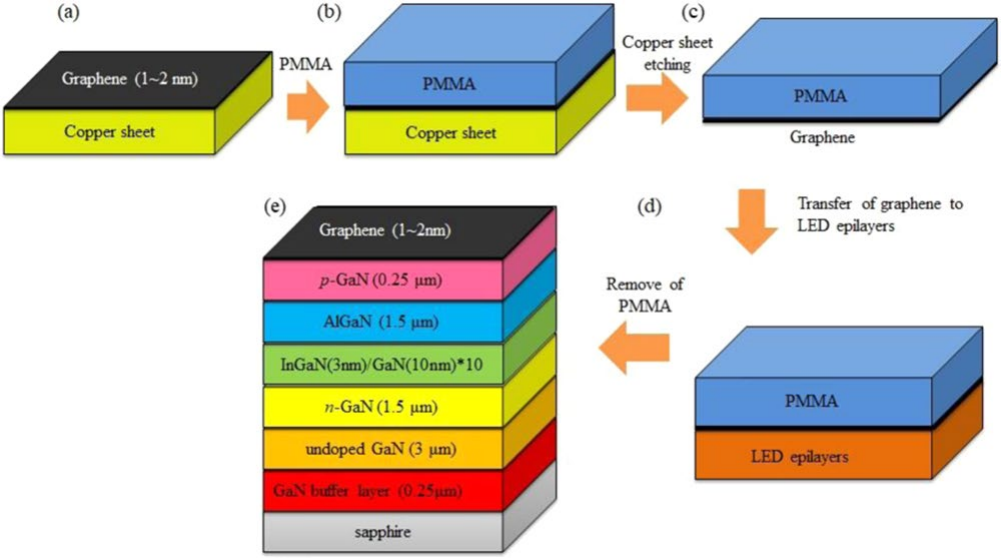
Fabrication processes of the InGaN/GaN MQW LEDs with graphene transparent conductive electrodes on the *p*-GaN layer: (**a**) Graphene was grown on a copper sheet by a chemical vapor deposition (CVD). (**b**) Poly(methyl methacrylate) (PMMA) was spin-coated on the graphene/copper sheet. (**c**) The copper sheet was etched in a 1 wt. % (NH_4_)_2_S_2_O_8_ solution. (**d**) The graphene with PMMA was transferred onto the *p*-GaN layer of the InGaN/GaN MQW LEDs. (**e**) The PMMA layer was removed by acetone solution.

Fig. 1 shows the fabrication procedures of the InGaN/GaN MQW LEDs with the graphene transparent conductive electrodes onto the *p*-GaN layer: (a) Graphene was grown on a copper sheet by a chemical vapor deposition (CVD). (b) Poly (methyl methacrylate) (PMMA) was spin-coated on the graphene/copper sheet. (c) The copper sheet was etched in a 1 wt. % (NH_4_)_2_S_2_O_8_ solution^11^. (d) The graphene with PMMA was transferred onto the *p*-GaN layer of the InGaN/GaN LEDs. (e) The PMMA layer was removed by acetone solution.

## Results and Discussion

### Device characteristics and carrier transport properties

#### EL studies

**Figure 2:**
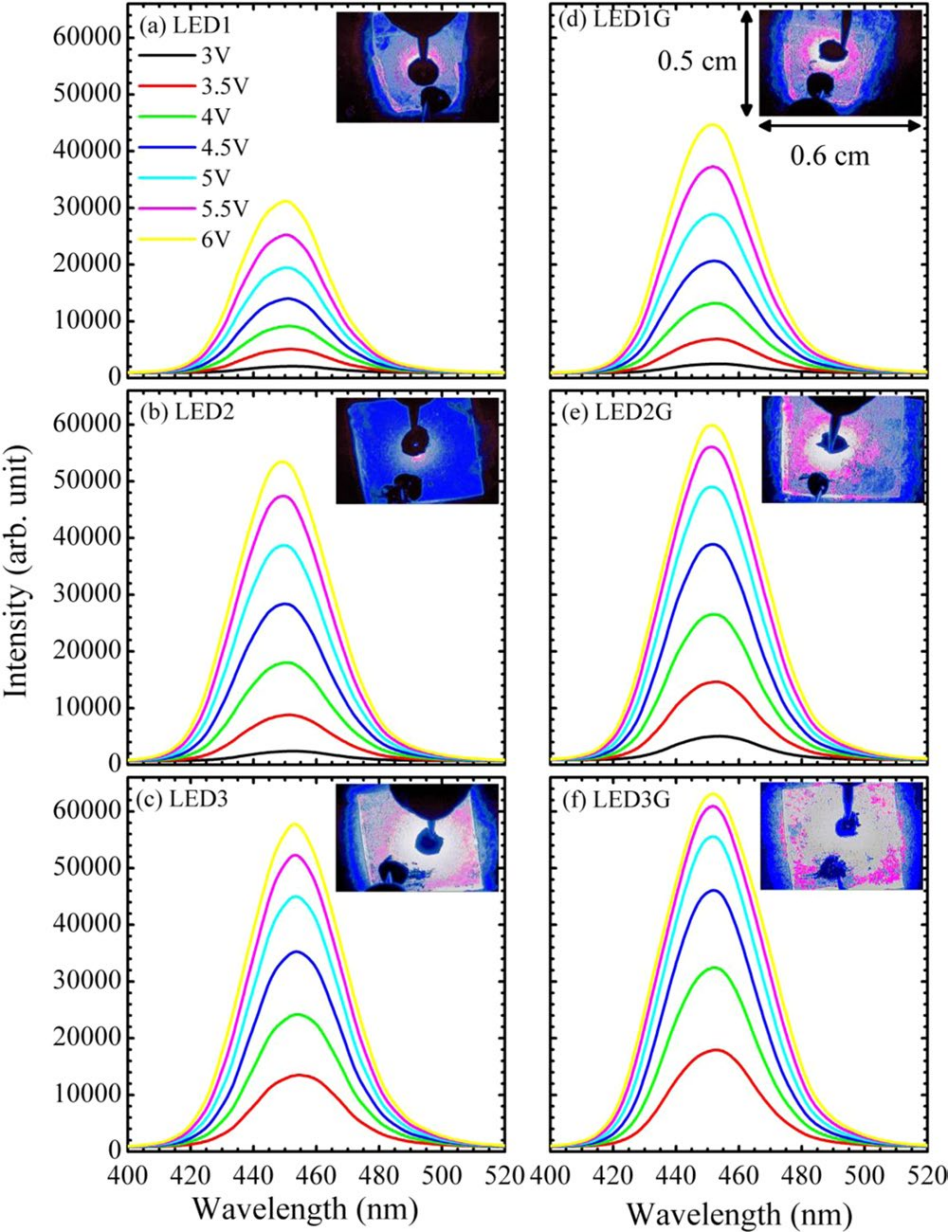
EL spectra for the (**a**) LED_1_, (**b**) LED_2_, (**c**) LED_3_, (**d**) LED_1G_, (**e**) LED_2G_, and (**f**) LED_3G_ samples. Insets of (**a–f**) show the EL images of the six LEDs operated under 3.0 V CW applied voltage, respectively.

Figure 2(a–f) show the room-temperature EL spectra of three LED samples (LED_1_, LED_2_, and LED_3_) and their counterparts with grapheme electrodes (LED_1G_, LED_2G_, and LED_3G_, respectively). Each inset in Fig. 2 shows the corresponding EL image of the LED operated under 3.0 V continuous-wave (CW) applied voltage. The luminescence areas for the LED_1_, LED_2_, and LED_3_ samples are brighter near the upper positive electrode. For nitride semiconductors, the electron mobility is fast than hole mobility^1,2^. With a higher electron mobility, the electrons transport more quickly into the MQWs near the positive electrode and wait for holes to recombine. Once the slow-moving holes transport and are captured into MQWs, electron-hole pairs will recombine and generate luminescence. In addition, stronger EL intensities and larger luminescence area of the LED_1G_, LED_2G_, and LED_3G_ samples show that graphene exhibits a good current spreading characteristic to distribute the injection current more uniformly.

**Figure 3:**
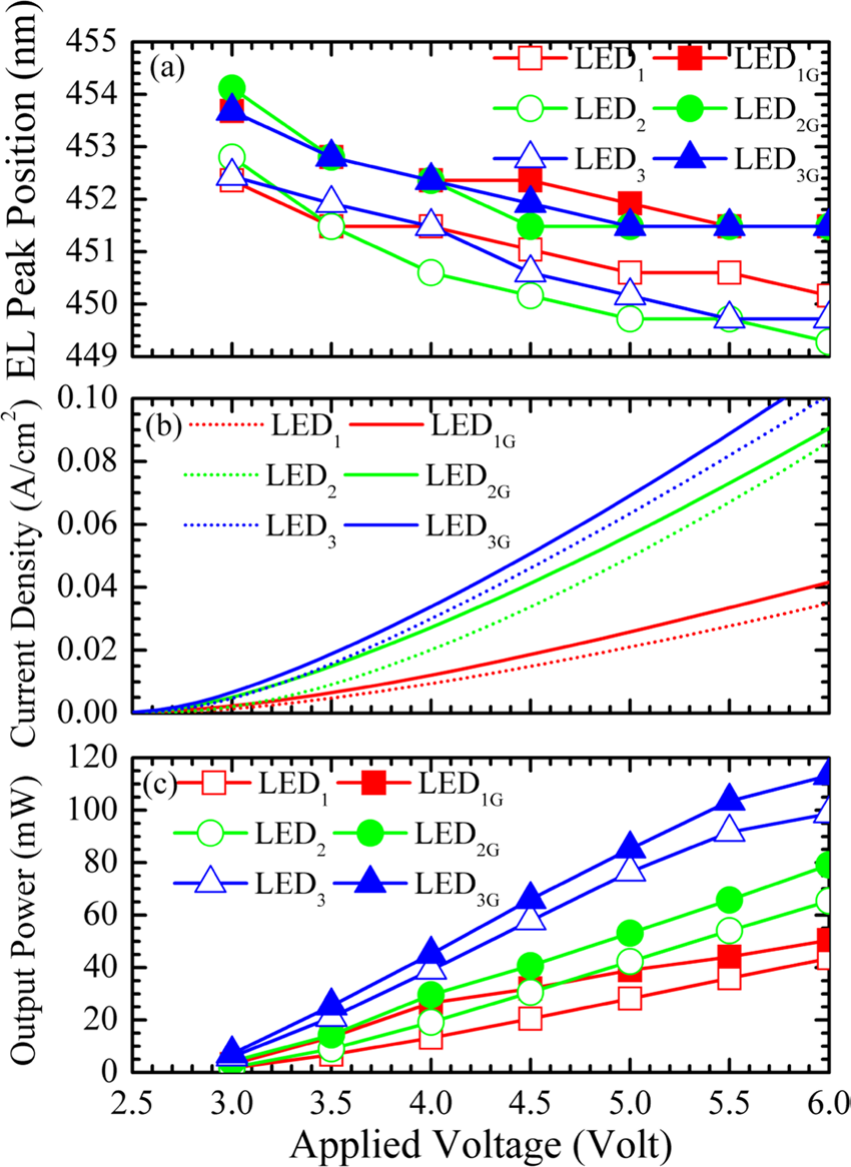
(**a**) EL peak position, (**b**) current density (*I*), and (**c**) output power as a function of applied voltage (*V*) for the LED_1_, LED_2_, LED_3_, LED_1G_, LED_2G_, and LED_3G_ samples.

Figure 3(a) shows the EL peak position as a function of applied voltage for the three LED samples and their counterparts with grapheme electrodes. With graphene transparent conductive electrodes, the EL peak positions of the LED_1G_, LED_2G_, and LED_3G_ samples are all red-shifted. Because of indium aggregation or phase separation in InGaN alloys, the EL emissions come from the recombination of localized excitons in In-rich InGaN clusters which formed spatial potential fluctuations and localized states for trapping carriers^1–6^. As shown later, the current density of the LED_1G_, LED_2G_, and LED_3G_ samples are all larger than those of the LED_1_, LED_2_, and LED_3_ samples, respectively. With a good spreading characteristic of graphene, more carriers can more efficiently transport into spatially distributed potential minimums (deeply localized states) for recombination^20–22^. Hence, the red-shifted EL peak positions of the LED_1G_, LED_2G_, and LED_3G_ samples are observed.

#### Current density and output power studies

Figure 3(b,c) show the current density (denoted as *I*) and output power as functions of applied voltage *V* for the LED_1_ versus(vs.) LED_1G_, LED_2_ vs. LED_2G_, and LED_3_ vs. LED_3G_ samples, respectively. The currents of the LED_1G_, LED_2G_, and LED_3G_ samples are all larger than those of the LED_1_, LED_2_, and LED_3_ samples, respectively. Although the graphene/GaN contact resistance and work function difference are both large, the current spreading characteristic of graphene distributes more current to inject into active region and would not degrade the electrical characteristics. The largest current density of the LED_3G_ sample implies that the LED_3G_ has the best graphene transparent conductive electrode as well as the best sample quality. Also, when the injected current is increased and the carriers are over populated. Hence, a blue-shift EL peak was observed in Fig. 3(a). In addition, the more injected carriers in the LED_1G_, LED_2G_, and LED_3G_ samples contribute to more output power. This characteristics shows that graphene acting as transparent conductive electrodes in InGaN/GaN MQW LEDs can improve device performance. From the better performance of LED_1G_, LED_2G_, and LED_3G_ samples, the bonding between LED and graphene should be good.

#### TREL study

**Figure 4:**
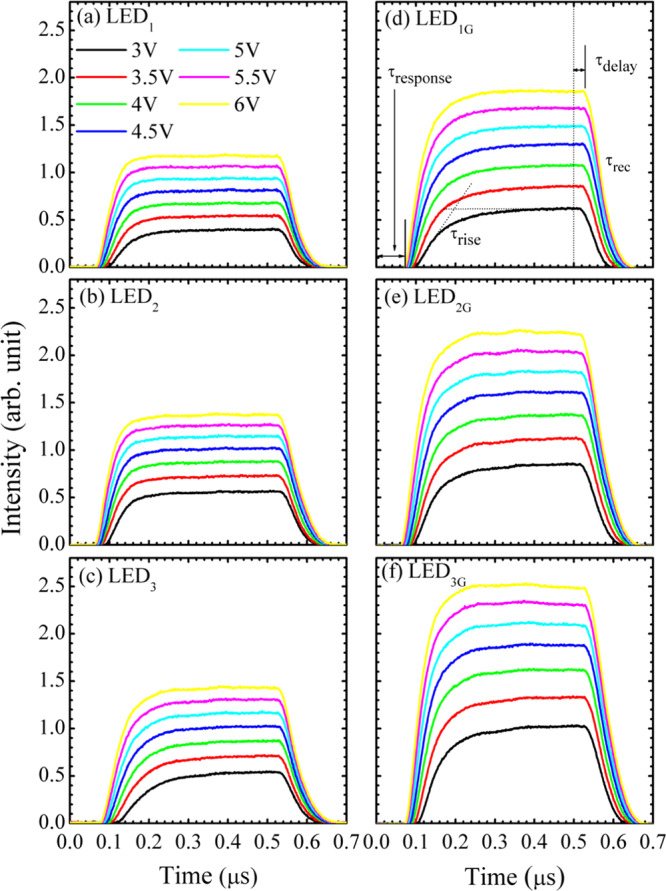
TREL profiles for the samples (**a**) LED_1_, (**b**) LED_2_, (**c**) LED_3_, (**d**) LED_1G_, (**e**) LED_2G_, and (**f**) LED_3G_ samples at room temperature. Response $({\tau }_{{\rm{resp}}})$, rise $({\tau }_{{\rm{rise}}})$, delay $({\tau }_{{\rm{delay}}})$, and recombination $({\tau }_{{\rm{rec}}})$ times are shown in (**d**).

Figure 4(a–f) show the TREL transit profiles of the LED_1_, LED_2_, LED_3_, LED_1G_, LED_2G_, and LED_3G_ samples, respectively. A stronger intensity, shorter response time, and steeply rising of the LED_1G_, LED_2G_, and LED_3G_ samples imply a better carrier transport and carrier capture efficiency^23^. In the TREL results, the response time (denoted as ${\tau }_{{\rm{resp}}}$) is extracted as the time interval between the start of input a short-pulse voltage signal and the emergence of the output PL light signal. The rising time ${\tau }_{{\rm{rise}}}$ is obtained by fitting the slope of the PL signal consecutive the response time. Subsequently, the PL signal reaches a steady-state to a maximum value. After the switch-off the input voltage signal, the PL signal will elapse a time interval (defined as the delay time ${\tau }_{{\rm{delay}}}$) before it exotically decays. Finally, the recombination time ${\tau }_{{\rm{rec}}}$ is obtained by fitting the exponential-decay profile of the PL signal.

**Figure 5:**
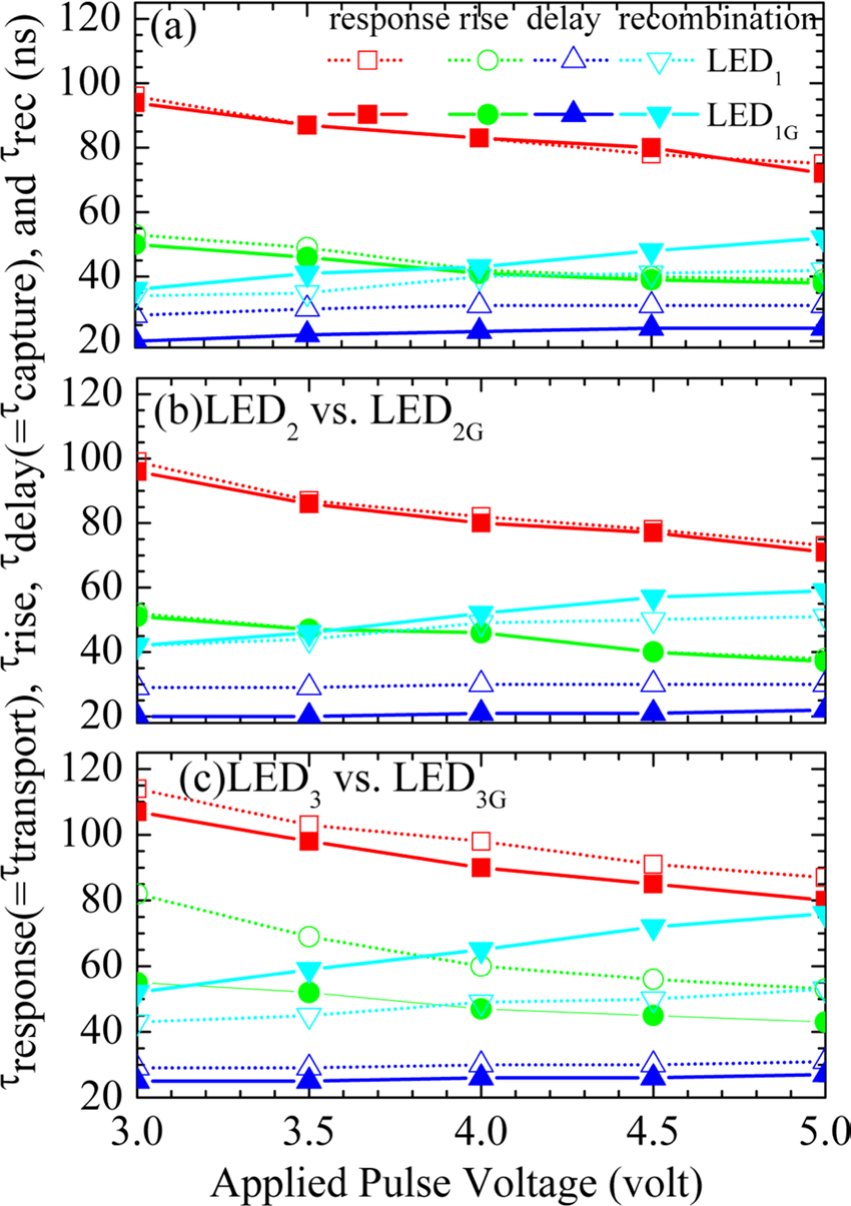
Response (=transport) $({\tau }_{{\rm{response}}}={\tau }_{{\rm{ransport}}})$, rise $({\tau }_{{\rm{rise}}})$, delay(=capture) $({\tau }_{{\rm{delay}}}={\tau }_{{\rm{capture}}})$, and recombination $({\tau }_{recombination})$ times as a function of applied pulse voltage for the (**a**) LED_1_ versus LED_1G_, (**b**) LED_2_ versus LED_2G_, and (**c**) LED_3_ versus LED_3G_ samples.

Figure 5(a–c) show the ${\tau }_{{\rm{resp}}}$, ${\tau }_{{\rm{rise}}}$, ${\tau }_{{\rm{delay}}}$, and ${\tau }_{{\rm{rec}}}$ as a function of applied pulse voltage for the LED_1_ vs. LED_1G_, LED_2_ vs. LED_2G_, and LED_3_ vs. LED_3G_ samples, respectively. As shown in Fig. 5, shorter ${\tau }_{{\rm{resp}}}$ and τ_rise_ of the LED_1G_, LED_2G_, and LED_3G_ samples imply that a good current spreading characteristic of graphene transparent conductive electrodes enhances carrier transport and carrier injection efficiency^23^. A shorter ${\tau }_{{\rm{delay}}}$ of the LED_1G_, LED_2G_, and LED_3G_ samples implies a better capture efficiency. For the LED_1G_, LED_2G_, and LED_3G_ samples, more carrier injection need more time for carrier recombination, resulting in a longer ${\tau }_{{\rm{rec}}}$. Among the LED_1G_, LED_2G_, and LED_3G_ samples, the most decreasing trend of ${\tau }_{{\rm{resp}}}$ and ${\tau }_{{\rm{rise}}}$ and also the most increasing trend of ${\tau }_{{\rm{rec}}}$ imply that the LED_3G_ sample has the best sample quality as well as the best graphene transparent conductive electrode quality. Therefore, the LED_3G_ sample exhibits the best efficiency of carrier transport among the three LED_G_ samples. These results will be further illustrated and verified by the following theoretical model.

#### 2-*N* theoretical model

In order to discuss the different time scales of carrier dynamics, a 2-*N* theoretical model was proposed to investigate the effects of graphene transparent conductive electrodes on the carrier transport, capture, and escape processes in the MQWs^23^. The rate equation of 2-*N* theoretical model is given by

1 $$\frac{d{N}_{{\rm{b}}}}{dt}=\frac{I}{e}-\frac{{N}_{{\rm{b}}}}{{\tau }_{{\rm{b}}}}-\frac{{N}_{{\rm{b}}}}{{\tau }_{{\rm{cap}}}}+\frac{{N}_{{\rm{w}}}}{{\tau }_{{\rm{esc}}}}$$

2 $$\frac{d{N}_{{\rm{w}}}}{dt}=\frac{{N}_{{\rm{b}}}}{{\tau }_{{\rm{cap}}}}-\frac{{N}_{{\rm{w}}}}{{\tau }_{{\rm{esc}}}}-\frac{{N}_{{\rm{w}}}}{{\tau }_{{\rm{w}}}}$$

where ${N}_{{\rm{b}}}$ and ${N}_{{\rm{w}}}$ are the carrier numbers in the barriers and quantum wells, respectively. ${\tau }_{{\rm{b}}}$, ${\tau }_{{\rm{cap}}}$, ${\tau }_{{\rm{esc}}}$, and ${\tau }_{{\rm{w}}}$ are carrier lifetime in the barrier, carrier capture time into the quantum wells, carrier escape time from the quantum wells, and carrier lifetime in the quantum wells, respectively^23^. The ${\tau }_{{\rm{cap}}}$, ${\tau }_{{\rm{esc}}}$, and ${\tau }_{{\rm{w}}}$ in the 2-*N* theoretical model can be approximately related the ${\tau }_{{\rm{resp}}}$, ${\tau }_{{\rm{delay}}}$, and ${\tau }_{{\rm{rec}}}$ in TREL data as follows:^23^

3 $${\tau }_{{\rm{resp}}}={\tau }_{{\rm{trans}}}$$

4 $${\tau }_{{\rm{delay}}}={\tau }_{{\rm{cap}}}$$

5 $${\tau }_{{\rm{rec}}}={\tau }_{{\rm{w}}}$$

It should be noted that the ${\tau }_{{\rm{resp}}}$ obtained from the TREL and the ${\tau }_{{\rm{trans}}}$ defined in the 2-*N* model roughly characterize the time span for carriers moving from the contact to the unconfined states in quantum-well region, while the ${\tau }_{{\rm{delay}}}$ obtained from the TREL and the ${\tau }_{{\rm{cap}}}$ defined in the 2-*N* model describe the time span for carriers transiting from the unconfined (bulk) states to the confined (quantum) states in the quantum-well region. A more detailed discussion on the physical meanings of ${\tau }_{{\rm{trans}}}$ and ${\tau }_{{\rm{cap}}}$ in the 2 N model was given in the previous work and will not be reiterated here for simplicity^23^.

Figure 5(a–c) show the ${\tau }_{{\rm{trans}}}$, ${\tau }_{{\rm{cap}}}$, and τ_rec_ as a function of applied pulse voltage for the LED_1_ vs. LED_1G_, LED_2_ vs. LED_2G_, and LED_3_ vs. LED_3G_ samples, respectively. The effects of graphene transparent conductive electrodes on the carrier transport, capture, and recombination dynamics of InGaN/GaN MQW LEDs can be well revealed from these results. With the graphene transparent conductive electrodes, the transport times ${\tau }_{{\rm{trans}}}$ of the LED_1G_ and LED_2G_ samples are slightly shorter in comparison with those of the samples without graphene. Meanwhile, the transport times ${\tau }_{{\rm{trans}}}$ of the LED_3G_ sample, due to its superior graphene quality, is much shorter than that of its without-graphene counterpart. In addition, a short transport time ${\tau }_{{\rm{trans}}}$ will also expedite carrier capture processes into the QWs, and thus result in a shorter capture time ${\tau }_{{\rm{cap}}}$ for the LED_1G_, LED_2G_, and LED_3G_ samples. In other word, due to the graphene electrode, more carriers transport in a larger luminescence area and consequently are captured more efficiently into the QWs. It should be noted that the response time obtained from the TREL and the transport time defined in the 2 N model roughly characterize the time span for carriers moving from the contact to the quantum-well region. Therefore, the injection efficiency is inversely proportional to the transport time^23^. As more carriers transport and are captured into QWs, more carriers will recombine in the QWs and have a longer recombination time ${\tau }_{{\rm{rec}}}$, in particular for the LED_3G_ sample.

### Discussion

**Figure 6:**
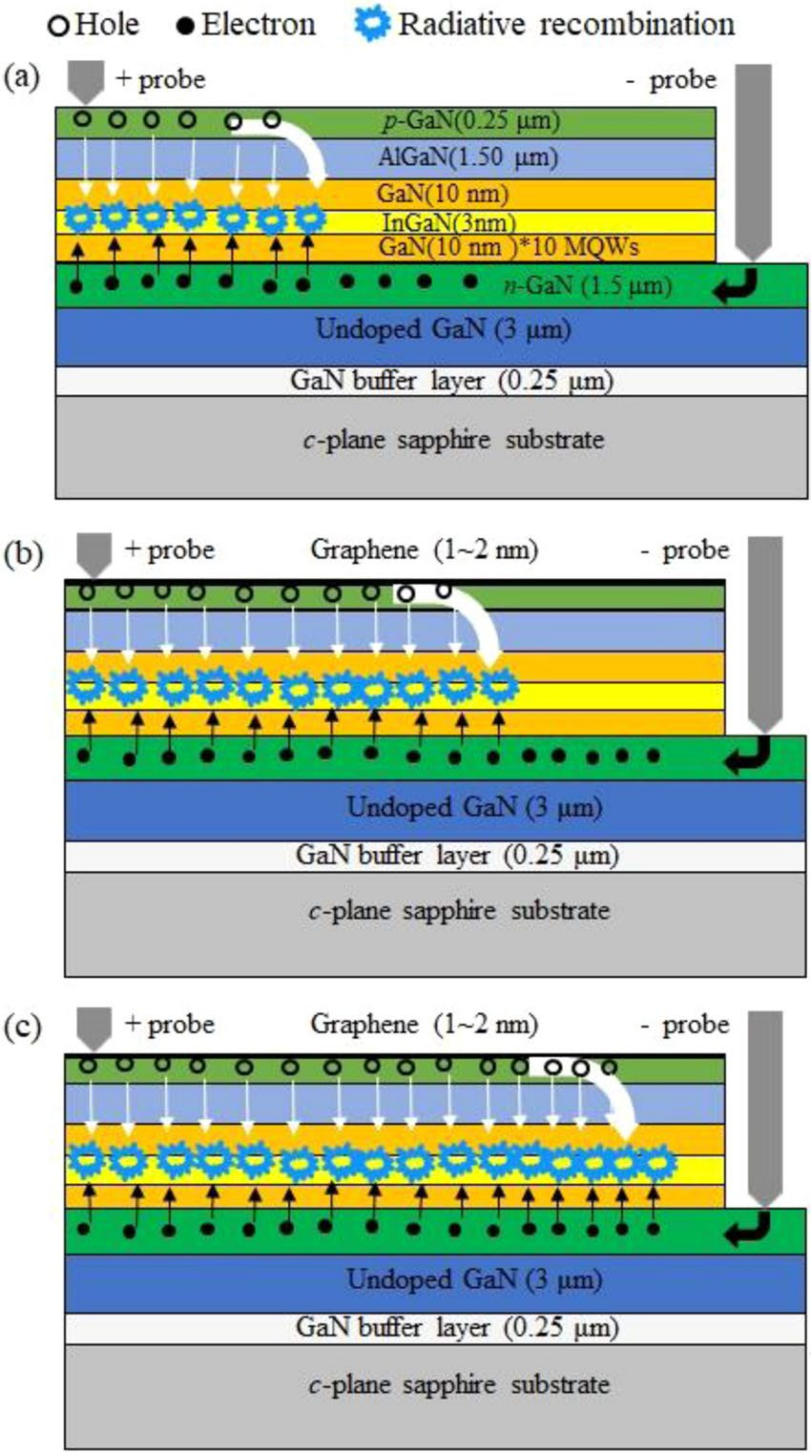
Schematic diagrams of the carrier transport, capture, and recombination processes in GaN/GaN MQW LEDs (**a**) without a graphene electrode, (**b**) with a graphene electrode, and (**c**) with a graphene electrode of better film quality. In the diagram (**a**), without a graphene electrode, a low hole injection efficiency into the MQWs lead to a limited current flow and low electron-hole recombination efficiency. The diagram (**b**) shows that the graphene electrode will enhance the hole injection efficiency into the MQWs and thus increase electron-hole recombination efficiency. In the diagram (**c**), as more carriers transport in a larger luminescence area, they will be more efficiently captured into the quantum wells and thus enhance electron-hole recombination.

Based on the above observations, carrier injection, carrier transport, carrier capture, and recombination dynamic schemes were proposed in Fig. 6 for explaining the improvement of device performance in LEDs with graphene transparent conductive electrodes. In Fig. 6(a), for nitride semiconductors, electron mobility is higher than hole mobility^1,2^. When the holes and electrons were injected into the MQWs, due to their higher electron mobility, electrons will transport and be captured more efficiently into to the MQWs than holes. As a result, the *p*-junction becomes the dominant factor in determining the overall carrier injection and recombination efficiency.

As shown in Fig. 6(b), the application of a graphene electrode in the *p*-junction will expedite the transport processes of holes along the lateral direction and thus resulting a broader current spreading and a larger luminescence area. As schematically illustrated in Fig. 6(c), if holes transport more efficiently, they can have a better chance to be captured into the QWs and contribute a higher carrier injection. As more carriers injected into the QWs, they will results in a longer recombination process. As a result, the LED_1G_, LED_2G_, and LED_3G_ samples have shorter transport and capture times, but longer recombination time than the LED_1_, LED_2_, and LED_3_ samples. The graphene transparent conductive electrodes can expedite carrier transport processes, shorten the transport time ${\tau }_{{\rm{trans}}}$, and increase the injection efficiency of holes. They also enhance carrier capture processes, shorten the capture time ${\tau }_{{\rm{cap}}}$, and consequently enhance the electron-hole recombination processes (which result in a longer recombination time ${\tau }_{{\rm{rec}}}$). In addition, the graphene contact will enhance carrier transport and thus shorten the ${\tau }_{{\rm{trans}}}$; therefore, more carriers will reach and accumulate in the quantum-well region. A larger carrier concentration needs a longer recombination time to consume these carries via radiative recombination processes in quantum wells. As a result, the recombination time will increase with graphene contacts. The effects of graphene transparent conductive electrodes on the carrier transport, carrier capture, and recombination dynamics of InGaN/GaN MQW LEDs can be well explained from the results of the TREL measurements and the analysis of the 2-*N* theoretical model.

## Conclusions

In this work, InGaN/GaN MQW LEDs with and without graphene transparent conductive electrodes are studied with I-V, EL, and TREL measurements. The results demonstrate that the applications of graphene electrodes on LED devices will spread injection carriers more uniformly into the active region and therefore result in a larger current density, broader luminescence area, and stronger EL intensity. In addition, the TREL data will be further analyzed by employing a 2-*N* theoretical model of carrier transport, capture, and escape processes. The combined experimental and theoretical results clearly indicate that those LEDs with graphene transparent conductive electrodes at *p*-junctions will have a shorter hole transport time along the lateral direction and thus a more efficient current spreading and thus a larger luminescence area. A shorter hole transport time will also expedite hole capture processes and thus result in a short capture time. Furthermore, as more carrier injected into the active regions of LEDs, thanks to graphene transparent conductive electrodes, excessive carriers need more time to proceed carrier recombination processes in QWs and thus result in a longer carrier recombination time. The research results provide important information for the carrier transport, carrier capture, and recombination processes in InGaN/GaN MQW LEDs with graphene transparent conductive electrodes.

## Supplementary information

Supplementary information.
