# Supplementary material for: Enhancing carrier transport and carrier capture with a good current spreading characteristic via graphene transparent conductive electrodes in InGaN/GaN multiple-quantum-well light emitting diodes
Source: Sci Rep. 2020 Jun 29;10:10539. doi: 10.1038/s41598-020-67274-1 (PMC7324381; doi:10.1038/s41598-020-67274-1)
Supplement: Supplementary file 1 — Supplementary information. [file 41598_2020_67274_MOESM1_ESM.docx]

**Enhancing carrier transport and carrier capture with a good current spreading characteristic** **via graphene transparent** **conductive electrodes in InGaN/GaN multiple-quantum-well light emitting diodes**

Shih-Wei Feng,^*1^ Ying-Hsiang Wang,^1^ Chin-Yi Tsai,^1^ Tzu-Huan Cheng,^2^ and Hsiang-Chen Wang^3^

*^1^Department of Applied Physics, National University of Kaohsiung*

*No.700, Kaohsiung University Road, Nan-Tzu Dist., 811. Kaohsiung, Taiwan, R.O.C.*

***^2^****LiveStrong Optoelectronics Cooperation, Kaohsiung, Taiwan, R.O.C.*

***^3^****Department of Mechanical Engineering and Advanced Institute of Manufacturing with High-tech Innovations, National Chung Cheng University, Chia-yi, Taiwan, R.O.C.*

*Corresponding authors: [*swfeng@nuk.edu.tw*](mailto:swfeng@nuk.edu.tw)

In our experimental process of transferring graphene to sample, we usually observe the number of graphene growth layers on copper foil through a microscope, as shown below. When graphene is transferred to PMMA, we will not perform measurement and verification.


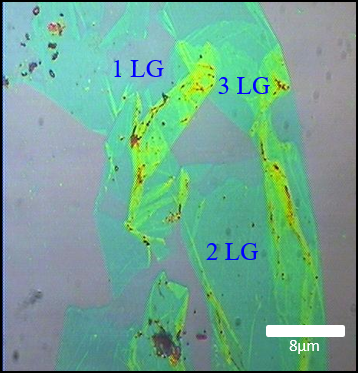


Figure 1. Observing the number of graphene growth layers on copper foil through a microscope.

We also have observed the Raman spectrum of the InGaN/GaN MQWs LED with few-layer graphene. The results were shown below. Measurement was performed 30 times at the known positions of the graphene layers. The Raman shift of the graphene film on a LED surface was mainly located at 1582 (G-band) and 2673 cm^−1^ (2D-band). On the G-band, the Raman signal strength increased with the number of layers, due to the larger number of carbon atoms present. The 2D-band, however, showed a decreasing intensity as the number of layers increased in agreement with previous reports^1^.

1. Y. Wang, Z. H. Ni, T. Yu, Z. X. Shen, H. M. Wang, Y. H. Wu, W. Chen and A. T. S. Wee, J. Phys. Chem., 2008, 112, 10637.


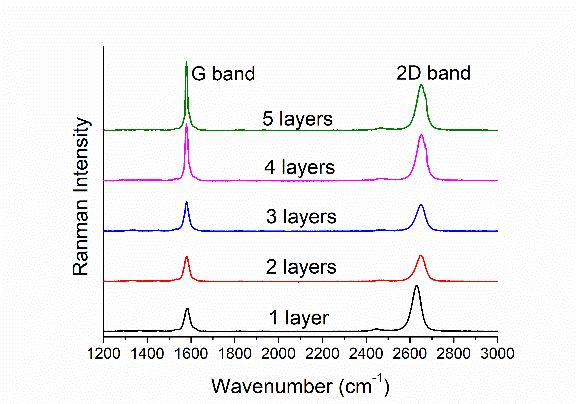


Figure 2 The Raman spectrum of the InGaN/GaN MQWs LED with few-layer graphene.

Besides optical microscopic images and microRaman spectral imaging, AFM measurement was conducted. But it is too hard to indicate the accurate numbers of layers without other characterizations. We got only one AFM image that is shown as following:


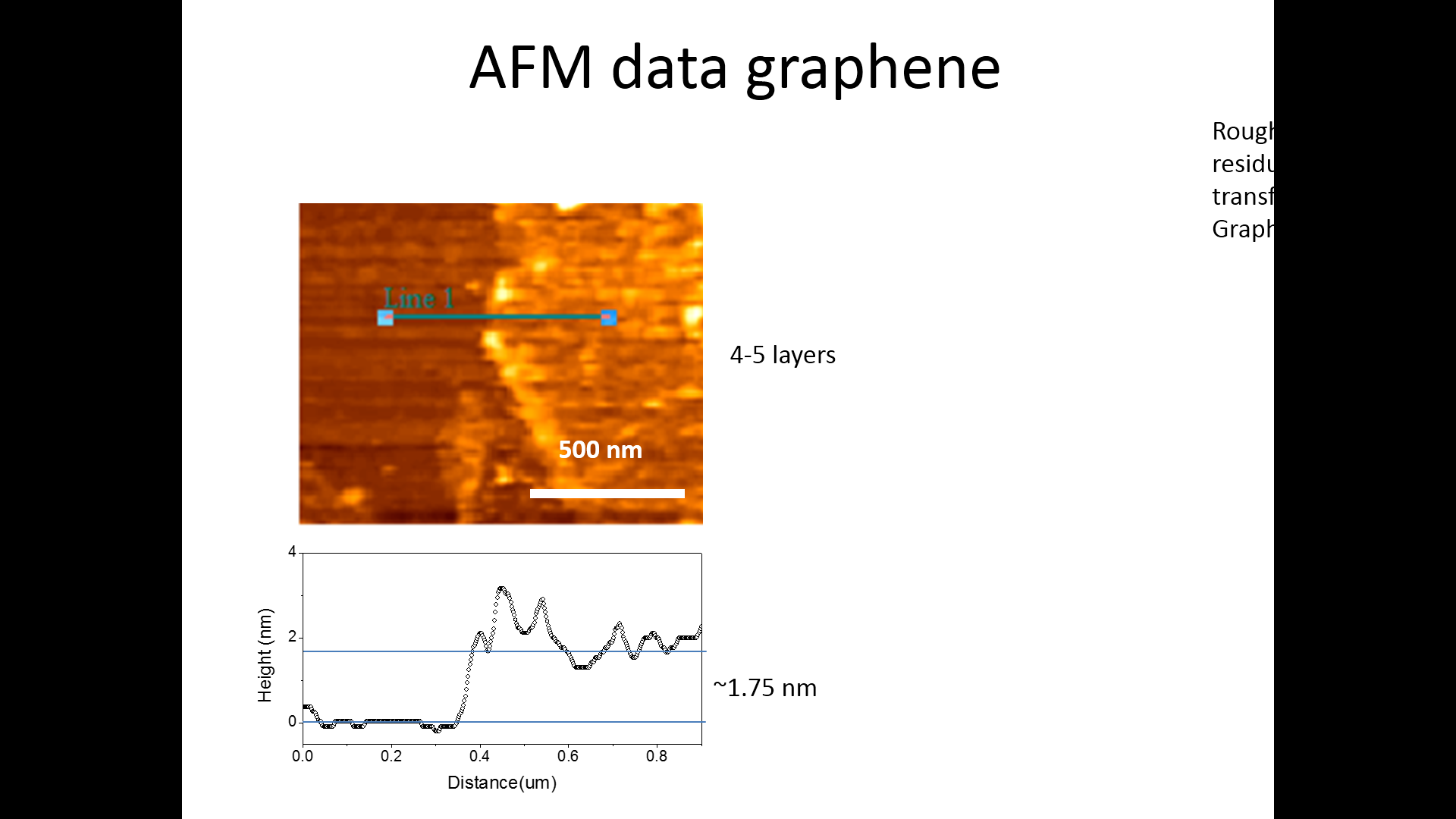


Figure 3 AFM image of graphene.

In this study, we have successfully measured the number of graphene layers on the LED using Raman spectroscopy and AFM, so we can prove that we have successfully transferred a few layers of graphene to the LED. From the results of AFM and Raman measurements, the quality of the graphene sheet is good. The graphene thickness has been corrected to be 1-2 nm.
